# Topical Application of Wogonin Provides a Novel Treatment of Knee Osteoarthritis

**DOI:** 10.3389/fphys.2020.00080

**Published:** 2020-02-18

**Authors:** Jacob F. Smith, Evan G. Starr, Michael A. Goodman, Romney B. Hanson, Trent A. Palmer, Jonathan B. Woolstenhulme, Jeffery A. Weyand, Andrew D. Marchant, Shawen L. Bueckers, Tanner K. Nelson, Matthew T. Sterling, Brandon J. Rose, James P. Porter, Dennis L. Eggett, David L. Kooyman

**Affiliations:** ^1^Department of Physiology and Developmental Biology, Brigham Young University, Provo, UT, United States; ^2^Statistics, Brigham Young University, Provo, UT, United States

**Keywords:** osteoarthritis, wogonin, inflammation, NF-κB, TGF-β1, HTRA1, MMP-13

## Abstract

Osteoarthritis (OA) is a degenerative joint disease characterized by inflammatory degradation of articular cartilage and subchondral bone. Wogonin, a compound extracted from the plant *Scutellaria baicalensis* (colloquially known as skullcap), has previously been shown to have direct anti-inflammatory and antioxidative properties. We examined the pain-reducing, anti-inflammatory, and chondroprotective effects of wogonin when applied as a topical cream. We validated the efficacy of delivering wogonin transdermally in a cream using pig ear skin in a Franz diffusion system. Using a surgical mouse model, we examined the severity and progression of OA with and without the topical application of wogonin. Using a running wheel to track activity, we found that mice with wogonin treatment were statistically more active than mice receiving vehicle treatment. OA progression was analyzed using modified Mankin and OARSI scoring and direct quantification of cyst-like lesions at the chondro-osseus junction; in each instance we observed a statistically significant attenuation of OA severity among mice treated with wogonin compared to the vehicle treatment. Immunohistochemistry revealed a significant decrease in protein expression of transforming growth factor β1 (TGF-β1), high temperature receptor A1 (HTRA1), matrix metalloprotease 13 (MMP-13) and NF-κB in wogonin-treated mice, further bolstering the cartilage morphology assessments in the form of a decrease in inflammatory and OA biomarkers.

## Introduction

Osteoarthritis (OA) is a chronic disease characterized by cartilage degradation, joint pain, decreased joint function, and diminished quality of life. Due to the nature and implications of this disease, the Federal Drug Administration has recently classified OA as a serious disease, placing it in the same category as cancer and cardiovascular disease (FDA, 2018). Risk factors include advanced age, genetic predisposition, and obesity (Coggon et al., 2001). It is predicted that as the populace ages, and with obesity trends significantly increasing, it is likely that a rise in OA prevalence will follow (Helmick et al., 2008; Lawrence et al., 2008). There is currently no known cure for OA; current pharmaceuticals such as NSAIDS or selective COX-2 inhibitors only serve to alleviate the pain, and because they have no beneficial effect on cartilage health they are not considered to be adequate disease-modifying drugs (Smith et al., 2016; Rafanan et al., 2018). Given the severity of the disease, a treatment is necessary that will alleviate, arrest, or reverse the progression of the disease.

Once dismissed as the result of passive and irreversible wear, the pathogenesis of OA is now better understood to be characterized by dysregulation of inflammatory and apoptotic (Hwang and Kim, 2015) pathways; maintaining a careful balance between anti-inflammatory and pro-inflammatory molecules is integral to joint health. Among the markers and effectors known to play a role in dysregulated joint inflammation are nuclear factor kappa-light-chain-enhancer of activated B cells (NF-κB) (Rigoglou and Papavassiliou, 2013), transforming growth factor β1 (TGF-β1) (Huang et al., 2018), high temperature requirement protein A-1 (HTRA1) (Holt et al., 2012), matrix metalloprotease-13 (MMP-13) (Rose and Kooyman, 2016), along with other matrix-degrading enzymes that are produced by chondrocytes and contribute to the degradation of the articular cartilage. Blocking such effectors to promote maintenance of joint homeostasis is one viable option to treat OA (Hu et al., 2016). However, many, if not all, of these inflammatory linked effectors have diverse functions and complete systemic or even local inhibition could lead to significant consequences in other tissues or organ systems.

Drug treatments for OA are divided into two categories: symptom modifying OA drugs (SYMOAD), which focus on ameliorating the discomfort associated with OA; or disease modifying OA drugs (DMOAD), which are drugs administered with the purpose of inhibiting cartilage degradation (Watt and Gulati, 2017). The current array of SYMOADs include non-steroidal anti-inflammatory drugs (NSAIDs), steroids, and narcotics. Each of these come with their own complicated set of side effects, preventing their use as long-term pharmacological agents (Watt and Gulati, 2017). With the limitations these options present, DMOADs emerge as the hope of an effective treatment. Wogonin, a compound extracted from the *Scutellaria baicalensis* plant, exhibits high anti-inflammatory (Huang X. et al., 2017; Khan et al., 2017) and antioxidative properties (Chow et al., 2012). In addition, it is known to have an inhibitory effect on pathways known to be inappropriately upregulated in OA, such as WNT (He et al., 2013; Usami et al., 2016; Stampella et al., 2017), and NF-κB (Nakamura et al., 2003; Xu et al., 2018); it is also known that wogonin has an activating effect on the nuclear factor-erythroid 2 (Zhong et al., 2013; Kim et al., 2016), an endogenous antioxidant defense mechanism known to protect against osteoarthritis (Guo et al., 2017). Previous work has demonstrated that after exposure to IL-1β, 1 μM wogonin exhibited a significant reduction in MMP-3 in rabbit articular chondrocytes (Park et al., 2015). Park et al. (2015), also pre-treated rat knees with intra-articular injection of 50 and 100 μM wogonin prior to injection of IL-1β and observed a significant reduction of MMP-3 expression. The properties of wogonin combined with previous studies formed a basis for its potential use as a DMOAD. Delivery of a DMOAD directly to an affected joint is an excellent treatment modality since it potentially mitigates effects on other tissues. While no current licensed DMOADs exist, the present study aims to assess the potential of wogonin in topical form, applied directly to the joint, as a DMOAD.

## Materials and Methods

### Mice and Joint Destabilization Procedure

Nine week-old C57B6 mice, randomized for sex, were acquired for this study and randomized for treatment (*n* = 9) and non-treatment (*n* = 11). Mice were anesthetized using VetOne Fluriso Isoflurane USP gas through a Somnosuite Kent Scientific small animal anesthesia system. The skin surrounding the right knee joint was prepped by clipping the fur and washing with a Vedco Veradine Providone-Iodine surgical scrub followed by VetOne Chlorohexidine Gluconate Antiseptic. The procedure was performed under a Wild Heerbrugg 355110 (Wild Heerbrugg AG, Switzerland) surgical microscope using sterile technique. The medial meniscal ligament was exposed by blunt dissection and subsequently transected using a number 11 scalpel to allow for displacement (Siebert et al., 2015). The joint capsule and skin were both closed following visual confirmation of destabilization using 7-0 absorbable Vicryl suture (Ethicon, Inc., Somerville, NJ, United States). These procedures were conducted under the protocol 160501 approved by the Brigham Young University Institutional Animal Care and Use Committee (IACUC), a committee, required by U.S. law that is recognized and authorized with oversight by the U.S. Office of Laboratory Animal Welfare. To adhere to the mandate of reducing animal numbers in research, the IACUC required that previous reports by us (Larkin et al., 2013) and others (Xu et al., 2010) suffice for sham surgery and no surgery controls. Mice were individually housed in standard open-top cages equipped with a voluntary running wheel with 1/8′′ corn cobb bedding. A 12-h light–dark cycle was used, with lights turning on at 6 A.M. Mice had *ad libitum* access to standard chow (LabDiet 5001) and water.

### Wheel Data

After intervention via DMM surgery, mice were isolated in separate cages equipped with a running wheel to track activity for 4 weeks. 20 μL treatment cream (TC) was applied to the right knees of mice in the treatment group every third day for 28 days. The TC applied to treated mice contained a 10 μM wogonin concentration and other natural compounds to aid in permeation (*n* = 9; five males, four females). The TC applied to untreated mice was an identical sham cream (SC) not containing wogonin (*n* = 11; seven males, four females). To allow the wogonin time to take full effect, only running wheel data from the final 14 days of the experiment was used in the statistical analysis.

### Tissue Processing

28 days post-DMM operation, animals were euthanized via carbon dioxide asphyxiation and cervical dislocation. The right knee of each was harvested and fixed in 4% paraformaldehyde. The subsequent decalcification procedure and embedding in paraffin wax were done according to the procedure reported (Siebert et al., 2015). Knees were sectioned at 6 μM thickness using a Heidelberg Microm microtome and the corresponding sections of tissue were stained with Safranin-O and Fast Green as previously described for histological analysis (Sheffield et al., 2018). Using a light microscope equipped with a digital camera, photographs of the stained joint tissue were taken at 10 and 20X magnification.

### Histological Analysis

To quantify the health of the joint, two slides of knee sections for each animal were selected and analyzed using the Modified Mankin and OARSI scoring system by two investigators who were blinded to the treatment received by each animal. Mankin scoring (Mankin et al., 1971) was done using a set of scores indicating severity of joint degradation, specifically cartilage erosion (0-6), chondrocyte periphery staining (0-2), spatial arrangement of chondrocytes (0-3), and background staining intensity (0-3) with 0 representing an unaffected joint and 6 representing severe OA. OARSI scoring was also completed (Glasson et al., 2010), which greater emphasized the cartilage depth and the special arrangements of the chondrocytes in the joints. Overall OARSI scoring was based on an osteoarthritic damage 0–6 subjective scoring system applied to all four quadrants of the knee.

### Cyst-Like Lesions (CLLs)

Cyst-like lesions are defined as a void space in the matrix at the chondro-osseus junction in the joint. Cysts in articular cartilage are one sign of OA progression in humans (Pritzker et al., 2006) and have been reported in OA-induced rats (Beckett et al., 2012). Due to CLL’s ability to disrupt the integrity of the articular cartilage matrix, they were included in our study as a measure of OA progression. Tissue sample sections stained with Safranin-O and Fast Green were visualized, and pictures were taken at 10 and 20X. CLLs were then counted blind as to treatment for each of the representative slides using the method explained in the listed protocol by two investigators who were blinded to the treatment received by each animal (Zhang et al., 2017).

### Immunohistochemistry Analysis

Immunohistochemistry (IHC) was performed on slides representative of serial sections of mouse knee joints from all animals. Separate slides were stained with antibodies against HTRA1, MMP-13, NF-κB, and TGF-β1. Each slide was deparaffinized and then blocked with 5% bovine serum albumin for 1 h. Primary antibodies against HTRA1 (ab38611) (Abcam, Cambridge, MA, United States), MMP-13 (ab3012) (Abcam, Cambridge, MA, United States), TGF-β1 (ab92486) (Abcam, Cambridge, MA, United States) and NF-κB (ab16502) (Abcam, Cambridge, MA, United States) were used. All antibodies were applied to specimens, and incubated overnight at 4°C. On the second day, samples were rinsed with PBS and then incubated with an avidin/biotin ABC mix (Vectastain elite ABC Kit). Slides were rinsed again with PBS and incubated with a species appropriate biotinylated goat anti rabbit secondary antibody. After a third rinse, a color reaction was initiated using a peroxidase substrate (Vector Labs, NovaRED). Negative controls were prepared by staining without the addition of primary antibody. Differences in staining intensity were compared qualitatively with treatment group controls. Blind counting of stained cells was performed using ImageJ (NIH, Bethesda, MD, United States). The n values for the different stains are as follows: HTRA1: TC *n* = 6 and SC *n* = 9, MMP-13: TC *n* = 7 and SC *n* = 9, TGF-β1: TC *n* = 7 SC *n* = 10, NF-κB: TC *n* = 6 and SC *n* = 7.

### ImageJ Analysis

The expression of levels of HTRA1, MMP-13, TGF-β1, and NF-κB were analyzed in a quantitative analysis by calculating the percentage of positive staining cells for each of the biomarkers in the joint in a defined 1200 × 280 pixel area of articular cartilage distal to the tibial plateau. Cell counting was then performed by two investigators who were blinded to the treatment received by each animal. The number of animal sections used in each stain group are previously reported in the IHC methods.

### Transdermal Cream Design and Use

The mouse knee was treated to transdermally deliver wogonin. Prior to the first application, the knee was treated with Nair^®^ to remove all hair. Nair^®^ was reapplied as often as necessary to keep the knee free of hair. Wogonin (CAS 632-85-9 ≥ 98% by HPLC) (681670, Sigma, St. Louis, MO, United States) was formulated at the specified concentration of 10 μM in propylene glycol (PG) based upon previous work we have done (Smith et al., 2019). The cream design was based upon previously published work, to maximize skin penetration. It consisted of a mixture of oleic acid (91541, Sigma, St. Louis, MO, United States), methylsulfonylmethane (MSM) (PHR1346, Sigma, St. Louis, MO, United States), PG (PHR1051, Sigma, St. Louis, MO, United States), shea butter (REAL African Shea Butter Pure Raw Unrefined From Ghana “IVORY,” Amazon) and peppermint oil (77411, Sigma, St. Louis, MO, United States) 0.1% (Zhang et al., 2009; Ezaki et al., 2013; Gobel et al., 2016) containing 10 μM wogonin. Sham cream was the exact same constituents except for wogonin.

#### Transdermal Cream Efficacy Testing

*In vitro* permeation of wogonin through skin was measured in a Franz diffusion cell utilizing a piece of pig skin harvested from the inside of the ear. Fresh pig ears were obtained from a local abattoir for each Franz diffusion cell assay as previously described (Ng et al., 2010). Pig ear skin has a structure and thickness similar to human skin (Ng et al., 2010). Pig ears were washed, fat removed, and depilated prior to placement as the barrier in the Franz diffusion cell. A 200 μL sample of wogonin cream was applied to the pig skin and allowed to incubate until penetration. The buffer was sampled periodically and the concentration of wogonin in the buffer measured using ultra violet (UV) spectrophotometry as previously described (Gao et al., 2008). Known amounts of wogonin in buffer were used to develop a standard curve down to the level of 0.1 μM wogonin. Permeation efficiency was measured by UV spectrophotometry in Franz diffusion buffer.

#### Assessment of Pain

Pain assessment in mice employed the Brigham Young University rodent pain assessment Standard Operating Procedure (SOP) developed by the University Veterinarian, based upon American Veterinary Medical Association (AVMA) guidelines and approved by the University IACUC.

#### Statistical Analysis

Statistical analysis was performed by the BYU Department of Statistics through the SAS program using a mixed-models analysis of variance (ANOVA) with a *post hoc t*-test. The dependent variables were the OARSI, Mankin scores or biomarker staining for the knee as well as the running wheel rotation data and CLLs. The independent variables were the treatment received, with wogonin or a vehicle cream without wogonin. Resulting *p*-values of < 0.05 were considered significant.

#### Power Analysis

The sample size is based upon statistical power calculations, with a significance level of 0.05 and reliability (power) of 0.9. The power analysis output was: Analysis: *A priori*: Compute required sample size Input:

Effect size f = 0.5676471 α err prob = 0.05 Power (1 − β err prob) = 0.9 Number of groups = 2

Number of measurements = 4 Corr among rep measures = 0:31 Output V Non − centrality parameter

λ = 12.0207630 Critical F = 4.4939985 Numerator df = 1.0000000

Denominator df = 16.0000000 Total sample size = 18 Actual power = 0.9019401 Therefore, 9 would be the lowest possible number of animals per treatment group to use

## Results

### Wogonin Skin Permeation

A 1 mL aliquot from the 5 mL Franz diffusion cell buffer provided a peak with an area of 4.41818 at a retention time of 8.016, corresponding to wogonin. Using the formula 4.41818 + 0.212/159.186, provided in the HPLC software, we calculated 0.145 uM wogonin in the 5 mL buffer reservoir. Using a dilution factor of 25 (200 μL into five ml) we determined that the buffer contained 3.64 μM wogonin. Thus, the efficacy of skin penetration of wogonin was about 36%. Skin permeation increased to 64% when the Franz diffusion assay was extended to 24 h (data not shown).

### OA Progression

Mice treated with wogonin had a significant decrease in OARSI (*p* < 0.05) and Mankin (*p* < 0.01) scores compared to sham mice. As an additional measure of cartilage health, CLLs were counted. These lesions have recently been shown to be associated with cartilage degradation and are more prevalent in joints with OA [9]. Mice treated with wogonin had a significant decrease in average counts of CLLs present in articular cartilage (*p* < 0.01). These data suggest that wogonin limits the progression of OA compared to the control. We noted no differences in response to treatment from either male or female mice.

### Running Wheel Activity and Pain Perception

After destabilization of the medial meniscus (DMM) surgery, we placed the mice in wheel cages in order to determine the amount of activity they employed as OA developed. While measuring daily activity, we proceeded to apply either topical wogonin treatment, or the control vehicle. On average, we found that mice treated with wogonin had a significant increase in running wheel activity (*p* < 0.05), indicating a decrease in pain perception from the control mice.

### Wogonin Decreased Biomarkers Associated With OA and Inflammation

Cell counting revealed significant decreases in biomarkers associated with OA and inflammation in mice treated with wogonin. HTRA1 (*p* < 0.001) and MMP-13 (*p* < 0.01), biomarkers associated with cartilage degradation, were decreased in wogonin mice. This is one potential explanation for a decrease in OA severity noted above. TGF-β1 (*p* = 0.05) and NF-κB (*p* < 0.05), factors associated with inflammation and OA progression, were also decreased in wogonin mice.

### Wogonin Decreased Biomarkers Associated With OA and Inflammation

Cell counting revealed significant decreases in biomarkers associated with OA and inflammation in mice treated with wogonin. HTRA1 (SC = 50%, TC = 10%; *p* < 0.001) and MMP-13 (SC = 28%, TC = 8%; *p* < 0.01), biomarkers associated with cartilage degradation, were decreased in wogonin mice. This is one potential explanation for a decrease in OA severity noted above. TGF-β1 (SC = 42%, TC = 34%; *p* = 0.05) and NF-κB (SC = 21%, TC = 4%; *p* < 0.05), factors associated with inflammation and OA progression, were also decreased in wogonin mice.

## Discussion

### Stability of Wogonin

The short and long-term stability of wogonin has previously been demonstrated in a variety of *in vivo* bioavailability and pharmacokinetic studies (Jian-chun et al., 2011; Zhang et al., 2013). We observed no dermal toxicity in our study. A previous study applying a higher concentration of wogonin similarly demonstrated no dermal toxicity (Kim et al., 2013).

### Wogonin Dose

Wogonin has previously been shown to affect the expression of MMP-1, MMP-13, and ADAMTS-4 at doses as low as 1 μM (Park et al., 2015). We targeted the dosage of wogonin for this work at 10 mM as a mid-level dose that takes into account efficiency of transdermal penetration. While other doses and pharmacokinetic studies may be appropriate in the future, our study provides a proof of concept for topical application of wogonin to treat OA.

### Wogonin on Joint Health

**FIGURE 1:**
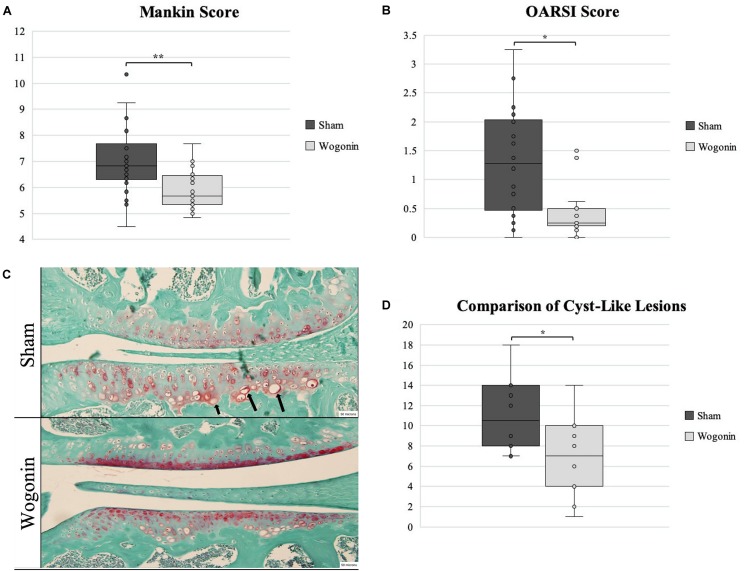
Tissues harvested from sham and wogonin mice were analyzed using OARSI and Mankin scoring systems and CLL counts. **(A,B)** OARSI and Mankin scoring revealed a significant decrease in OA severity in wogonin mice. **(C)** Safranin-O staining of tissues used for OARSI and Mankin scoring and CLL counting. Arrows indicate typical CLLs. **(D)** Wogonin mice had significantly less CLLs present in articular cartilage than sham mice. ^∗^*p* < 0.05; **p < 0.01.

**FIGURE 2:**
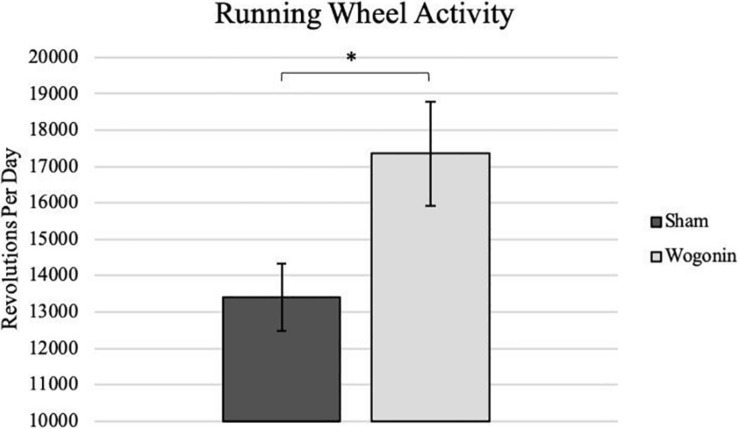
Average daily activity as recorded on a running wheel by sham and wogonin mice. After DMM surgery and concurrent with treatment, all mice were housed in individual wheel cages that measured daily activity. On average, wogonin mice ran significantly more than sham mice. *p < 0.05.

**FIGURE 3:**
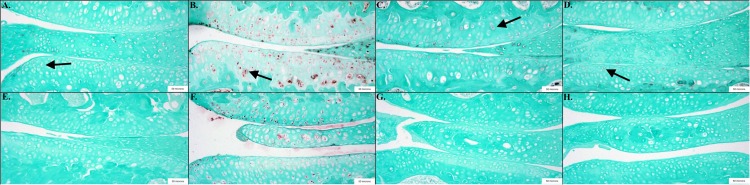
Representative images showing the results of the immunohistochemical and histological staining performed to analyze the presence of OA biomarkers. Mice treated with wogonin showed significant decreases in the percentage of cells stained positive compared to controls, including NF-κB *p* < 0.05, *n* = 6 (TC), 9 (SC); TGF-β1 *p* = 0.05, *n* = 7 (TC), 10 (SC); HTRA1 *p* < 0.001, *n* = 6 (TC), 9 (SC) and MMP-13 *p* < 0.01, *n* = 7 (TC), 9 (SC). **(A)** Sham Cream NF-κB; **(B)** Sham Cream TGF-β1; **(C)** Sham Cream HTRA1; **(D)** Sham Cream MMP-13; **(E)** Treatment Cream NF-κB; **(F)** Treatment Cream TGF-β1; **(G)** Treatment Cream HTRA1; **(H)** Treatment Cream MMP-13. Arrows indicate representative positively stained cells.

Both the sham and treatment creams used in this study were identical, with the exception of wogonin. This includes the presence of the menthol, recognized as both an analgesic and counterirritant on the U.S. Federal Drug Administration (FDA) DIA-OTC ingredient list (FDA, 2019). As such it is listed as the active ingredient by many SYMOAD products. We conclude, that application of wogonin in a topical cream significantly prevents joint damage after knee destabilization surgery (see Figures 1–3). The decrease in OARSI and Mankin scores compared to those receiving a vehicle control indicate that overall joint health was preserved by topical application of wogonin. A significant decrease in the presence of CCLs in treated knees following destabilization surgery is further indication of the ability of wogonin to prevent joint degradation.

Topical application of the compound wogonin, proves the safest and most efficient mechanism for delivery. The LD_50_ of wogonin administered I.V. is 286.15 mg/kg (Qi et al., 2009), many fold higher than mice experienced in this study. Systemic treatment of NSAIDs, via oral pill or supplement, increases the risk of adverse effects, specifically gastrointestinal discomfort, and decreases the effective impact of the drug in comparison to its topical application (Klinge and Sawyer, 2013). Additionally, topical application allows for targeted delivery to the location of interest without compromising other bodily processes through unintended interactions. Another common delivery approach for OA requires the use of injections into the joint capsule. This mechanical disruption of the tissues can induce further complications and even compound the damage of osteoarthritis and cartilage degeneration (Gonzalez-Fuentes et al., 2010). Additionally, the avascular environment of cartilage increases the difficulty of delivering nutrients or treatment to the chondrocytes through the bloodstream. While wogonin has proven effective and safe for systemic use (Huang D. S. et al., 2017), we believe that the ideal delivery method for the treatment of OA is as a topical cream, paired with other compounds to increase permeation and diffusion into the joint capsule.

Previous work has demonstrated that after exposure to IL-1β, 1 μM wogonin exhibited a significant reduction in MMP-3 in rabbit articular chondrocytes (Park et al., 2015). We observed a similar response as Park et al. (2015), when we used doses of wogonin on human chondrocytes (SCC042, Sigma-Aldrich, St. Louis, MO, United States) to titrate wogonin dose response to IL-1β exposure. Park et al. (2015), also pre-treated rat knees with intra-articular injection of 50 and 100 μM wogonin prior to injection of IL-1β and observed a significant reduction of MMP-3 expression.

While further research and human clinical trials are needed to establish this compound as an appropriate DMOAD, this study, using an *in vivo* mouse model, shows that wogonin has great potential in this regard. The following sections discuss potential mechanisms by which wogonin exerts its chondroprotective effects on the joint.

### Wogonin and Reactive Oxygen Species

Reactive oxygen species (ROS) are involved in many intracellular and extracellular processes associated with OA, including inflammatory signaling, apoptotic mechanisms, and extracellular degradation (Lepetsos and Papavassiliou, 2016). Oxidative stress is caused by an overload of ROS and the decreased ability of a cell to trap and contain free radicals. Oxidative stress is implicated in the activation of the MAPK (Touyz and Schiffrin, 2004) and NF-κB pathways (D’Angio and Finkelstein, 2000), which have been described as integral mediators of OA pathology. Wogonin has been shown to be a powerful free radical scavenger (Gao et al., 1999), leading to an overall decrease in oxidative stress and subsequent inhibition of mechanisms leading to OA. Wogonin has also been shown to upregulate nuclear factor (erythroid-derived 2)-like 2 (NRF2), a transcription factor whose downstream effects lead to the upregulation of enzymes that combat the negative effects of oxidative stress (Khan et al., 2017). The ability to scavenge free radicals and activate NRF-2 makes wogonin a powerful antioxidative agent. It is possible that these antioxidative properties of wogonin are a major mechanism by which we see an increase in overall joint health compared to the control. Work is ongoing in our lab to elucidate the effects that wogonin has on mitigating oxidative stress *in vivo*.

### Wogonin on the Primary Cilium

We have previously reported that wogonin affects BBS3 expression (Smith et al., 2019), known to traffic proteins to primary cilia (Su et al., 2014). The primary cilium is known to play a critical role in proper cartilage development, including mediation of chondrogenesis (Buscher et al., 1997) and development of planar cell polarity. The importance of these early developmental processes to adult homeostasis is apparent from deformation of articular cartilage in mutant animals with non-functional ciliary components intraflagellar transport protein-88 (IFT-88) (Chang et al., 2012), Polaris (McGlashan et al., 2007), divers Bardet–Biedl syndrome (BBS) proteins (Kaushik et al., 2009) and early-onset osteoarthritis in these mutants. It has been found that pathways controlled by the primary cilium in early development such as sonic hedgehog (SHH) (Stott and Chuong, 1997) and WNT signaling (Stampella et al., 2017) are dysregulated in osteoarthritis, alluding to a role of an as of yet uncharacterized failure of ciliary function in the development of osteoarthritis. As an inflammatory disease it is known that the transcription factor NF-κB plays a significant role in coordinating chondrocyte responses to inflammatory stimuli (Saito and Tanaka, 2017). We show in this study that wogonin has the effect of diminishing the expression of NF-κB in osteoarthritic chondrocytes (see Figure 3).

It has been demonstrated that primary cilia promote ubiquitination and proteasomal degradation of active NF-κB through the activity of inhibitor of κB kinase (IKK) (Wann et al., 2014). It is also known that wogonin inhibits the phosphorylation of IκB and IKKα/β, processes documented to be coordinated by the primary cilium (Yao et al., 2014). Owing to this data, we believe that the diminished NF-κB activity demonstrated in this and separate (Xu et al., 2017) instances caused by wogonin comes through a modulatory effect on ciliary signaling. In addition to this data, it is known that wogonin has been demonstrated in separate instances to have inhibitory activity on SHH (Owen et al., 2017) and WNT signaling (Song et al., 2015), processes which are upregulated in osteoarthritis (Luyten et al., 2009; Chang et al., 2012) and mediated by the primary cilium. The fact that wogonin directly counters these pathways with strong connections to the primary cilia and possesses antiosteoarthritic activity strongly suggests that wogonin exerts some sort of corrective action on the primary cilia, and future research should focus on interactions between wogonin and ciliary homeostasis.

## Conclusion

Decreased expression of MMP-13, TGF-β1, NF-κB and HTRA1 in the joint, paired with fewer CLLs, lower OARSI and Mankin scores, with significantly greater joint use on the running wheel after knee destabilization surgery suggests that the treatment cream, containing wogonin, interrupts the important OA related pathways, and reduces inflammatory and catabolic markers. Thus, demonstrating a rescue of the arthritic joint from cartilage degradation while promoting homeostasis. This work does not demonstrate that topical application of wogonin has disease modifying properties in the sense that damage is reversed. Further studies will be required to assess disease reversal. Rather, the model used and experimental design show that topical application of wogonin does significantly slow or abrogate joint damage progression. Wogonin exhibits anti-inflammatory, anti-oxidative protective effects modulated, in part, through primary-cilium. A modest dose of wogonin in a topical cream promotes joint health in mice and represents a novel treatment for the disease of osteoarthritis.

### Strengths and Limitations

This paper demonstrates the DMOAD capability of topical application of wogonin. We know of no other such report for wogonin or any other topically applied agent. Some significant and important findings in this work are that wogonin appears to be able to cross the skin barrier and retain biological activity. This paper, like virtually all scientific studies, leaves some unanswered questions. For instance, if wogonin is doing more than just treating inflammation, what else might it be doing and through which metabolic pathways? Pharmacokinetic studies need to be performed regarding the topical application of wogonin, including dose optimization. Additionally, this work needs to be repeated in a larger animal model so that knee joint related tissues such as synovial fluid, fat pad and cartilage can be studied for both wogonin concentration and important biomarkers associated with OA.

## Patents

The topical formulation used in this study has U.S. and International patents pending.

## Data Availability Statement

The datasets generated for this study are available on request to the corresponding author.

## Ethics Statement

This study was performed in strict accordance with the recommendations in the U.S. Guide for the Care and Use of Laboratory Animals of the National Institutes of Health. All of the animals were handled according to approved Brigham Young University Institutional Animal Care and Use Committee (IACUC) protocol 16-0501. The BYU IACUC is overseen and approved with oversight by the U.S. Office of Laboratory Animal Welfare. Animals were housed according to IACUC recommendations. Methods of euthanasia used were carbon dioxide inhalation followed by cervical dislocation, or anesthesia induced by ketamine/xylazine followed by transcardial perfusion. Humane endpoints were strictly observed, and every effort was made to minimize suffering. Animal pain and discomfort was monitored using the Brigham Young University IACUC required Pain and Distress Monitoring scale, which conforms to the guidelines of the American Veterinary Medical Association.

## Institutional Safety

All U.S. National Institutes of Health guidelines for research involving Recombinant or Synthetic Nucleic Acid Molecules were followed. All chemical use was according to the U.S. Occupational Safety and Health Administration guidelines as overseen by the Brigham Young University Office of Risk Management.

## Author Contributions

JS, ES, BR, and JP provided direct oversight for the project. JS, ES, MG, RH, TP, JBW, JAW, AM, SB, TN, MS, and BR performed all the histological work including analyses associated therewith. DE was responsible for all statistical work assisted by JS and ES. DE also assisted with experimental design. DK conceived the project and was the overall coordinator. The manuscript was written by the combined efforts of all authors.

## Conflict of Interest

DK is the inventor of the patented topical wogonin cream used in this study and as such has financial interest in sales of the product. He did not receive any financial support from the commercial entity that licensed his patents nor did they play any role in the research project, including design and interpretation of results. He is the only author with any conflict of interest. The remaining authors declare that the research was conducted in the absence of any commercial or financial relationships that could be construed as a potential conflict of interest.

## References

[B1] Beckett J. Jin W. Schultz M. Chen A. Tolbert D. Moed B. R. (2012). Excessive running induces cartilage degeneration in knee joints and alters gait of rats. *J. Orthop. Res.* 30 1604–1610. 10.1002/jor.22124 22508407

[B2] Buscher D. Bosse B. Heymer J. Ruther U. (1997). Evidence for genetic control of Sonic hedgehog by Gli3 in mouse limb development. *Mech. Dev.* 62 175–182. 10.1016/s0925-4773(97)00656-4 9152009

[B3] Chang C. F. Ramaswamy G. Serra R. (2012). Depletion of primary cilia in articular chondrocytes results in reduced Gli3 repressor to activator ratio, increased Hedgehog signaling, and symptoms of early osteoarthritis. *Osteoarthritis Cartilage* 20 152–161. 10.1016/j.joca.2011.11.009 22173325 PMC3260404

[B4] Chow S. E. Chen Y. W. Liang C. A. Huang Y. K. Wang J. S. (2012). Wogonin induces cross-regulation between autophagy and apoptosis via a variety of Akt pathway in human nasopharyngeal carcinoma cells. *J. Cell. Biochem.* 113 3476–3485. 10.1002/jcb.24224 22689083

[B5] Coggon D. Reading I. Croft P. McLaren M. Barrett D. Cooper C. (2001). Knee osteoarthritis and obesity. *Int. J. Obes. Relat. Metab. Disord.* 25 622–627. 11360143 10.1038/sj.ijo.0801585

[B6] D’Angio C. T. Finkelstein J. N. (2000). Oxygen regulation of gene expression: a study in opposites. *Mol. Genet. Metab.* 71 371–380. 10.1006/mgme.2000.3074 11001829

[B7] Ezaki J. Hashimoto M. Hosokawa Y. Ishimi Y. (2013). Assessment of safety and efficacy of methylsulfonylmethane on bone and knee joints in osteoarthritis animal model. *J. Bone Miner. Metab.* 31 16–25. 10.1007/s00774-012-0378-9 23011466

[B8] FDA, (2018). *OA A Serious Disease.* Silver Spring, MD: FDA.

[B9] FDA, (2019). *DIA-OTC Drug List.* Silver Spring, MD: FDA.

[B10] Gao J. Sanchez-Medina A. Pendry B. A. Hughes M. J. Webb G. P. Corcoran O. (2008). Validation of a HPLC method for flavonoid biomarkers in skullcap (*Scutellaria*) and its use to illustrate wide variability in the quality of commercial tinctures. *J. Pharm. Pharm. Sci.* 11 77–87. 18445366 10.18433/j39g6v

[B11] Gao Z. Huang K. Yang X. Xu H. (1999). Free radical scavenging and antioxidant activities of flavonoids extracted from the radix of *Scutellaria baicalensis* Georgi. *Biochim. Biophys. Acta* 1472 643–650. 10.1016/s0304-4165(99)00152-x 10564778

[B12] Glasson S. S. Chambers M. G. Van Den Berg W. B. Little C. B. (2010). The OARSI histopathology initiative - recommendations for histological assessments of osteoarthritis in the mouse. *Osteoarthritis Cartilage* 18(Suppl. 3), S17–S23. 10.1016/j.joca.2010.05.025 20864019

[B13] Gobel H. Heinze A. Heinze-Kuhn K. Gobel A. Gobel C. (2016). Peppermint oil in the acute treatment of tension-type headache. *Schmerz* 30 295–308. 10.1007/s00482-016-0109-6 27106030

[B14] Gonzalez-Fuentes A. M. Green D. M. Rossen R. D. Ng B. (2010). Intra-articular hyaluronic acid increases cartilage breakdown biomarker in patients with knee osteoarthritis. *Clin. Rheumatol.* 29 619–624. 10.1007/s10067-010-1376-8 20101426

[B15] Guo Y. X. Liu L. Yan D. Z. Guo J. P. (2017). Plumbagin prevents osteoarthritis in human chondrocytes through Nrf-2 activation. *Mol. Med. Rep.* 15 2333–2338. 10.3892/mmr.2017.6234 28259976

[B16] He L. Lu N. Dai Q. Zhao Y. Zhao L. Wang H. (2013). Wogonin induced G1 cell cycle arrest by regulating Wnt/beta-catenin signaling pathway and inactivating CDK8 in human colorectal cancer carcinoma cells. *Toxicology* 312 36–47. 10.1016/j.tox.2013.07.013 23907061

[B17] Helmick C. G. Felson D. T. Lawrence R. C. Gabriel S. Hirsch R. Kwoh C. K. (2008). Estimates of the prevalence of arthritis and other rheumatic conditions in the United States, Part I. *Arthritis Rheum.* 58 15–25. 10.1002/art.23177 18163481

[B18] Holt D. W. Henderson M. L. Stockdale C. E. Farrell J. T. Kooyman D. L. Bridgewater L. C. (2012). Osteoarthritis-like changes in the heterozygous sedc mouse associated with the HtrA1-Ddr2-Mmp-13 degradative pathway: a new model of osteoarthritis. *Osteoarthritis Cartilage* 20 430–439. 10.1016/j.joca.2011.11.008 22155431

[B19] Hu H. Yang B. Li Y. Zhang S. Li Z. (2016). Blocking of the P2X7 receptor inhibits the activation of the MMP-13 and NF-kappaB pathways in the cartilage tissue of rats with osteoarthritis. *Int. J. Mol. Med.* 38 1922–1932. 10.3892/ijmm.2016.2770 27748894

[B20] Huang D. S. Yu C. Y. Wu C. H. Lin J. Y. (2017). Protective effects of wogonin against Alzheimer’s disease by inhibition of amyloidogenic pathway. *Evid. Based Complement. Alternat. Med.* 2017:3545169. 10.1155/2017/3545169 28680449 PMC5478820

[B21] Huang J. Zhao L. Chen D. (2018). Growth factor signalling in osteoarthritis. *Growth Factors* 36 187–195. 10.1080/08977194.2018.1548444 30624091 PMC6430655

[B22] Huang X. Wu H. Wang L. Zheng L. Zhao J. (2017). Protective effects of baicalin on rabbit articular chondrocytes in vitro. *Exp. Ther. Med.* 13 1267–1274. 10.3892/etm.2017.4116 28413465 PMC5377289

[B23] Hwang H. S. Kim H. A. (2015). Chondrocyte apoptosis in the pathogenesis of osteoarthritis. *Int. J. Mol. Sci.* 16 26035–26054. 10.3390/ijms161125943 26528972 PMC4661802

[B24] Jian-chun L. Fei-hu C. Hai-jun D. Shu G. (2011). Study on plasma concentration and bioavailability of wogonin in beagle’s dogs. *Chin. Herb. Med.* 3 144–149.

[B25] Kaushik A. P. Martin J. A. Zhang Q. Sheffield V. C. Morcuende J. A. (2009). Cartilage abnormalities associated with defects of chondrocytic primary cilia in Bardet-Biedl syndrome mutant mice. *J. Orthop. Res.* 27 1093–1099. 10.1002/jor.20855 19195025 PMC3845817

[B26] Khan N. M. Haseeb A. Ansari M. Y. Devarapalli P. Haynie S. Haqqi T. M. (2017). Wogonin, a plant derived small molecule, exerts potent anti-inflammatory and chondroprotective effects through the activation of ROS/ERK/Nrf2 signaling pathways in human Osteoarthritis chondrocytes. *Free Radic. Biol. Med.* 106 288–301. 10.1016/j.freeradbiomed.2017.02.041 28237856 PMC5490997

[B27] Kim E. H. Jang H. Shin D. Baek S. H. Roh J. L. (2016). Targeting Nrf2 with wogonin overcomes cisplatin resistance in head and neck cancer. *Apoptosis* 21 1265–1278. 10.1007/s10495-016-1284-8 27544755

[B28] Kim T. W. Song I. B. Lee H. K. Kim M. S. Ham S. H. Cho J. H. (2013). Assessment of dermal safety of *Scutellaria baicalensis* aqueous extract topical application on skin hypersensitivity. *Planta Med.* 79 959–962. 10.1055/s-0032-1328714 23818268

[B29] Klinge S. A. Sawyer G. A. (2013). Effectiveness and safety of topical versus oral nonsteroidal anti-inflammatory drugs: a comprehensive review. *Phys. Sportsmed.* 41 64–74. 10.3810/psm.2013.05.2016 23703519

[B30] Larkin D. J. Kartchner J. Z. Doxey A. S. Hollis W. R. Rees J. L. Wilhelm S. K. (2013). Inflammatory markers associated with osteoarthritis after destabilization surgery in young mice with and without Receptor for Advanced Glycation End-products (RAGE). *Front. Physiol.* 4:121. 10.3389/fphys.2013.00121 23755017 PMC3664783

[B31] Lawrence R. C. Felson D. T. Helmick C. G. Arnold L. M. Choi H. Deyo R. A. (2008). Estimates of the prevalence of arthritis and other rheumatic conditions in the United States, Part II. *Arthritis Rheum.* 58 26–35. 10.1002/art.23176 18163497 PMC3266664

[B32] Lepetsos P. Papavassiliou A. G. (2016). ROS/oxidative stress signaling in osteoarthritis. *Biochim. Biophys. Acta* 1862 576–591. 10.1016/j.bbadis.2016.01.003 26769361

[B33] Luyten F. P. Tylzanowski P. Lories R. J. (2009). Wnt signaling and osteoarthritis. *Bone* 44 522–527. 19136083 10.1016/j.bone.2008.12.006

[B34] Mankin H. J. Dorfman H. Lippiello L. Zarins A. (1971). Biochemical and metabolic abnormalities in articular cartilage from osteo-arthritic human hips. II. Correlation of morphology with biochemical and metabolic data. *J. Bone Joint Surg. Am.* 53 523–537. 10.2106/00004623-197153030-00009 5580011

[B35] McGlashan S. R. Haycraft C. J. Jensen C. G. Yoder B. K. Poole C. A. (2007). Articular cartilage and growth plate defects are associated with chondrocyte cytoskeletal abnormalities in Tg737orpk mice lacking the primary cilia protein polaris. *Matrix Biol.* 26 234–246. 10.1016/j.matbio.2006.12.003 17289363

[B36] Nakamura N. Hayasaka S. Zhang X. Y. Nagaki Y. Matsumoto M. Hayasaka Y. (2003). Effects of baicalin, baicalein, and wogonin on interleukin-6 and interleukin-8 expression, and nuclear factor-kappab binding activities induced by interleukin-1βeta in human retinal pigment epithelial cell line. *Exp. Eye Res.* 77 195–202. 10.1016/s0014-4835(03)00116-7 12873450

[B37] Ng S. F. Rouse J. J. Sanderson F. D. Meidan V. Eccleston G. M. (2010). Validation of a static Franz diffusion cell system for in vitro permeation studies. *AAPS PharmSciTech* 11 1432–1441. 10.1208/s12249-010-9522-9 20842539 PMC2974154

[B38] Owen H. C. Appiah S. Hasan N. Ghali L. Elayat G. Bell C. (2017). Phytochemical modulation of apoptosis and autophagy: strategies to overcome chemoresistance in leukemic stem cells in the bone marrow microenvironment. *Int. Rev. Neurobiol.* 135 249–278. 10.1016/bs.irn.2017.02.012 28807161

[B39] Park J. S. Lee H. J. Lee D. Y. Jo H. S. Jeong J. H. Kim D. H. (2015). Chondroprotective effects of wogonin in experimental models of osteoarthritis in vitro and in vivo. *Biomol. Ther.* 23 442–448. 10.4062/biomolther.2015.045 26336584 PMC4556204

[B40] Pritzker K. P. Gay S. Jimenez S. A. Ostergaard K. Pelletier J. P. Revell P. A. (2006). Osteoarthritis cartilage histopathology: grading and staging. *Osteoarthritis Cartilage* 14 13–29. 10.1016/j.joca.2005.07.014 16242352

[B41] Qi Q. Peng J. Liu W. You Q. Yang Y. Lu N. (2009). Toxicological studies of wogonin in experimental animals. *Phytother. Res.* 23 417–422. 10.1002/ptr.2645 19003942

[B42] Rafanan B. S. Jr. Valdecanas B. F. Lim B. P. Malairungsakul A. Tassanawipas W. Shiyi C. (2018). Consensus recommendations for managing osteoarthritic pain with topical NSAIDs in Asia-Pacific. *Pain Manag.* 8 115–128. 10.2217/pmt-2017-0047 29251544

[B43] Rigoglou S. Papavassiliou A. G. (2013). The NF-kappaB signalling pathway in osteoarthritis. *Int. J. Biochem. Cell Biol.* 45 2580–2584. 10.1016/j.biocel.2013.08.018 24004831

[B44] Rose B. J. Kooyman D. L. (2016). A tale of two joints: the role of matrix metalloproteases in cartilage biology. *Dis. Markers* 2016:4895050. 10.1155/2016/4895050 27478294 PMC4961809

[B45] Saito T. Tanaka S. (2017). Molecular mechanisms underlying osteoarthritis development: notch and NF-kappaB. *Arthritis Res. Ther.* 19:94. 10.1186/s13075-017-1296-y 28506315 PMC5433029

[B46] Sheffield I. D. McGee M. A. Glenn S. J. Baek D. Y. Coleman J. M. Dorius B. K. (2018). Osteoarthritis-like changes in bardet-biedl syndrome mutant ciliopathy mice (Bbs1(M390R/M390R)): evidence for a role of primary cilia in cartilage homeostasis and regulation of inflammation. *Front. Physiol.* 9:708 10.3389/fphys.2018.00708 PMC6018413 29971011

[B47] Siebert M. Wilhelm S. K. Kartchner J. Z. Mecham D. Reynolds P. R. Kooyman D. L. (2015). Effect of pharmacological blocking of TLR-4 on osteoarthritis in mice. *J. Arthritis* 4:164.

[B48] Smith J. F. Starr E. G. Goodman M. A. Hanson R. B. Palmer T. A. Woolstenhulme J. B. (2019). Topical application of wogonin provides a novel treatment of knee osteoarthritis. *FASEB J.* 33(Suppl. 1): 487.22. 10.3389/fphys.2020.00080 PMC7040489 32132930

[B49] Smith S. R. Deshpande B. R. Collins J. E. Katz J. N. Losina E. (2016). Comparative pain reduction of oral non-steroidal anti-inflammatory drugs and opioids for knee osteoarthritis: systematic analytic review. *Osteoarthritis Cartilage* 24 962–972. 10.1016/j.joca.2016.01.135 26844640 PMC4996269

[B50] Song X. Zhou Y. Zhou M. Huang Y. Li Z. You Q. (2015). Wogonin influences vascular permeability via Wnt/beta-catenin pathway. *Mol. Carcinog.* 54 501–512. 10.1002/mc.22093 24136474

[B51] Stampella A. Monteagudo S. Lories R. (2017). Wnt signaling as target for the treatment of osteoarthritis. *Best Pract. Res. Clin. Rheumatol.* 31 721–729. 10.1016/j.berh.2018.03.004 30509416

[B52] Stott N. S. Chuong C. M. (1997). Dual action of sonic hedgehog on chondrocyte hypertrophy: retrovirus mediated ectopic sonic hedgehog expression in limb bud micromass culture induces novel cartilage nodules that are positive for alkaline phosphatase and type X collagen. *J. Cell Sci.* 110(Pt 21), 2691–2701. 9427387 10.1242/jcs.110.21.2691

[B53] Su X. Driscoll K. Yao G. Raed A. Wu M. Beales P. L. (2014). Bardet-Biedl syndrome proteins 1 and 3 regulate the ciliary trafficking of polycystic kidney disease 1 protein. *Hum. Mol. Genet.* 23 5441–5451. 10.1093/hmg/ddu267 24939912 PMC4168828

[B54] Touyz R. M. Schiffrin E. L. (2004). Reactive oxygen species in vascular biology: implications in hypertension. *Histochem. Cell Biol.* 122 339–352. 10.1007/s00418-004-0696-7 15338229

[B55] Usami Y. Gunawardena A. T. Iwamoto M. Enomoto-Iwamoto M. (2016). Wnt signaling in cartilage development and diseases: lessons from animal studies. *Lab. Invest.* 96 186–196. 10.1038/labinvest.2015.142 26641070 PMC4838282

[B56] Wann A. K. T. Chapple J. P. Knight M. M. (2014). The primary cilium influences interleukin-1 beta-induced NF kappa B signalling by regulating IKK activity. *Cell. Signal.* 26 1735–1742. 10.1016/j.cellsig.2014.04.004 24726893 PMC4064300

[B57] Watt F. E. Gulati M. (2017). New drug treatments for osteoarthritis: what is on the horizon? *Eur. Med. J. Rheumatol.* 2 50–58. 30364878 PMC6198938

[B58] Xu L. Servais J. Polur I. Kim D. Lee P. L. Chung K. (2010). Attenuation of osteoarthritis progression by reduction of the discoidin domain receptor 2 in mice. *Arthritis Rheum.* 62 2736–2744. 10.1002/art.27582 20518074 PMC2946478

[B59] Xu P. P. Zuo H. Q. Zhou R. F. Chen B. Ouyang J. (2018). Wogonin inhibits growth of mantle cell lymphoma cells through nuclear factor-kappab signaling pathway. *Chin. Med. J.* 131 495–497. 10.4103/0366-6999.225064 29451160 PMC5830840

[B60] Xu X. Zhang X. Zhang Y. Yang L. Liu Y. Huang S. (2017). Wogonin reversed resistant human myelogenous leukemia cells via inhibiting Nrf2 signaling by Stat3/NF-kappaB inactivation. *Sci. Rep.* 7:39950. 10.1038/srep39950 28150717 PMC5288730

[B61] Yao J. Zhao L. Zhao Q. Zhao Y. Sun Y. Zhang Y. (2014). NF-kappaB and Nrf2 signaling pathways contribute to wogonin-mediated inhibition of inflammation-associated colorectal carcinogenesis. *Cell Death Dis.* 5:e1283. 10.1038/cddis.2014.221 24901054 PMC4611709

[B62] Zhang M. Wong I. G. Gin J. B. Ansari N. H. (2009). Assessment of methylsulfonylmethane as a permeability enhancer for regional EDTA chelation therapy. *Drug Deliv.* 16 243–248. 10.1080/10717540902896362 19538004

[B63] Zhang Z. J. Beckett J. Schon L. (2017). Cyst-like lesions at chondro-osseous junction. *Calcif. Tissue Int.* 101 549–552. 10.1007/s00223-017-0306-z 28725908

[B64] Zhang Z. Q. Liua W. Zhuang L. Wang J. Zhang S. (2013). Comparative pharmacokinetics of baicalin, wogonoside, baicalein and wogonin in plasma after oral administration of pure baicalin, radix scutellariae and scutellariae-paeoniae couple extracts in normal and ulcerative colitis rats. *Iran. J. Pharm. Res.* 12 399–409. 24250647 PMC3813259

[B65] Zhong Y. Zhang F. Sun Z. Zhou W. Li Z. Y. You Q. D. (2013). Drug resistance associates with activation of Nrf2 in MCF-7/DOX cells, and wogonin reverses it by down-regulating Nrf2-mediated cellular defense response. *Mol. Carcinog.* 52 824–834. 10.1002/mc.21921 22593043

